# History of the American Medical Association

**Published:** 1856-01

**Authors:** 


*History of the American Medical Association.*—Several months since, our Colleague, Prof. Johnson, briefly noticed the volume, the title to which is given below.—Lest some of our readers might suppose his notice was influenced by personal favor, we copy the following from the January number of the *American Journal of Medical Sciences*, over the initials of Dr. D. Francis Condie, of Philadelphia. Copies may be had by applying at the Publication Office of this Journal:—

"*History of the American Medical Association from its Organization up to January*, 1855. By N. S. DAVIS, M.D., Professor of Principles and Practice of Medicine, and Clinical Medicine in Rush Medical College, etc. etc. etc. *To which is appended Biographical Notices, with Portraits of the Presidents of the Association, and of the Author.* Edited by S. W. BUTLER, M.D. 8vo. pp. 191. Philadelphia: Lippincott, Grambo & Co., 1855.

"Whether the American Medical Association is destined to be a permanent institution, that, after having by perseverance accomplished the primary objects of its organization, shall continue a bond of union and of strength, for the protection of the rights and the furtherance of the interests of the medical profession throughout the United States—increasing in dignity, influence, and efficiency with each year that is added to its age, or whether from some unforeseen and unanticipated cause it shall, sooner or later, cease to exist, still must its organization and its doings ever form prominent and most interesting items in the history of our profession in this country.

"Long or short lived as the Association may be, it has already given a proper direction to many of the things that most deeply affect the prosperity of our profession, and has made upon it, throughout, an impression, the salutary influence of which cannot be easily or speedily counteracted.

"It is true that, in its attempts to work out the problem of reform in medical education, it has been encountered by contending and discordant interests, which it has not yet been able to conciliate; and yet, even in this department of its operations, facts incontestably prove that its efforts have not been without a beneficial result—far short, unquestionably, of what was anticipated from them, in the outset, but still sufficient to create the hope that, by steady perseverance, the Association may ultimately succeed in the entire accomplishment of all its plans of reform.

"Look upon it in whatever light we may—as an experiment destined to end in a total failure, or as an organization the operations of which have been so far beneficial, and are destined to become still more so in the future, the history of the American Medical Association is, and always will be, a subject of deep interest to the American physician, and we are therefore pleased that Dr. Davis, whose name is identified with the Association from its orign—has undertaken the task of recording its history, which he has accomplished with great ability and perfect impartiality.

"As the work of Dr. Davis has doubtless found its way, before this, into the hands of the major portion of our readers, it is unnecessary for us to enter into an examination of its contents. The author presents a plain, unvarnished narrative of the causes which led to the organization of the Association, and of its proceedings—the subjects discussed, the plans digested, and the recommendations and suggestions adopted, at its several sessions, from the preliminary meeting in 1846, to that of May, 1854, inclusive. In an appendix, short biographical notices are given of the eight gentlemen who have served as Presidents of the Association, and of its historian.

"We would say to those who have not yet become possessed of the work before us, to do so without delay, and give to it a careful perusal; for we are desirous that every medical man in the country should become acquainted with the character and doings of the Association, as we would wish to enlist every one on its behalf, as well for the general benefit of the profession at large, as for that of each of its members individually, and we know of no more certain means of effecting this than by promoting the circulation of Dr. Davis's history. D. F. C."

